# Surface Modification of Multi-Walled Carbon Nanotubes via Hemoglobin-Derived Iron and Nitrogen-Rich Carbon Nanolayers for the Electrocatalysis of Oxygen Reduction

**DOI:** 10.3390/ma10050564

**Published:** 2017-05-20

**Authors:** Wensheng Li, Lingtao Sun, Rong Hu, Wenli Liao, Zhongbin Li, Yanrong Li, Chaozhong Guo

**Affiliations:** 1 College of Chemistry and Chemical Engineering, Chongqing University, Chongqing 400044, China; liwensheng@cqu.edu.cn; 2 Research Institute for New Materials Technology, Engineering Research Center of New Energy Storage Devices and Applications, Chongqing University of Arts and Sciences, Chongqing 402160, China; ltsun@cqwu.edu.cn (L.S.); rhu@cqwu.edu.cn (R.H.); yrli@cqwu.edu.cn (Y.L.); 3 School of Materials and Chemical Engineering, Chongqing University of Arts and Sciences, Chongqing 402160, China; liaowenli@cqwu.edu.cn (W.L.); lzb@cqwu.edu.cn (Z.L.)

**Keywords:** oxygen reduction, electrocatalyst, active site, carbon nanotube, hemoglobin

## Abstract

The great challenge of boosting the oxygen reduction reaction (ORR) activity of non-noble-metal electrocatalysts is how to achieve effective exposure and full utilization of nitrogen-rich active sites. To realize the goals of high utilization of active sites and fast electron transport, here we report a new strategy for synthesis of an iron and nitrogen co-doped carbon nanolayers-wrapped multi-walled carbon nanotubes as ORR electrocatalyst (N-C@CNT-Fe) via using partially carbonized hemoglobin as a single-source precursor. The onset and half-wave potentials for ORR of N-C@CNT-Fe are only 45 and 54 mV lower than those on a commercial Pt/C (20 wt.% Pt) catalyst, respectively. Besides, this catalyst prepared in this work has been confirmed to follow a four-electron reaction mechanism in ORR process, and also displays ultra-high electrochemical cycling stability in both acidic and alkaline electrolytes. The enhancement of ORR activity can be not only attributed to full exposure and utilization of active site structures, but also can be resulted from the improvement of electrical conductivity owing to the introduction of CNT support. The analysis of X-ray photoelectric spectroscopy shows that both Fe–N and graphitic-N species may be the ORR active site structures of the prepared catalyst. Our study can provide a valuable idea for effective improvement of the electrocatalytic activity of non-noble-metal ORR catalysts.

## 1. Introduction

The development of low-cost and high-performance oxygen reduction electrocatalysts is a key solution for rapid commercialization of various metal-air batteries (MABs) and fuel cells (FCs) [1]. Today, the mainstream platinum (Pt)-based materials with the best oxygen reduction reaction (ORR) performance are subjected to scarce reserves, exorbitant price, and easy poisoning, which largely hinders their market-oriented applications [2]. The design and synthesis of cheap and resourceful non-noble-metal electrocatalysts to absolutely replace the Pt-based electrocatalyst becomes an ideal and effective solution [3]. In particular, lots of research efforts have mainly focused on iron–nitrogen–carbon (Fe–N–C) composite catalysts owing to their excellent ORR activity and stability [4]. Recently, many research groups interestingly prepared Fe–N–C or nitrogen-doped carbon (NC) catalysts by direct pyrolysis of biomass rich in various proteins as nitrogen and carbon sources [5,6,7,8]. However, it is found that the packing and agglomeration of NC-based catalysts decreases the surface density of N-doped active sites and further limits their effective utilization, significantly impeding the enhancement of the ORR electrocatalytic activity [9,10,11]. To solve these problems, we previously proposed an interesting method to prepare iron and nitrogen co-doped carbons-modified carbon nanospheres via utilization of a blood protein pyropolymer derived from pig blood as a simple Fe–N-enriched precursor [12,13]. This method can not only avoid the agglomeration of blood protein during pyrolysis process, but also enhance the electronic conductivity of the catalyst and expose more N-rich active sites on its surface, largely facilitating the fast electron-transport of the ORR.

Carbon nanotubes (CNT) possess high electronic conductivity, excellent mechanical flexibility and thermal/chemical stability, special one-dimensional hollow structure, and nano-channels, which can be widely used as a fast electron transfer channel and carbon support for Fe–N–C catalysts [9]. Besides, traditional bioproteins—e.g., hemoglobin (Hb)—have been widely exploited for a class of carbon and nitrogen sources in recent years [14]. Confessedly, Hb includes abundant hemes consisting of Fe atoms attached to a planar porphyrin-like structure [15]. Unfortunately, the produced carbonized material from direct heat-treatment of Hb at high temperature in an inert atmosphere can commonly display poor ORR activity in acidic medium, because most of inner active sites do not play an important role in the ORR electrocatalysis process [9,14]. For this reason, the increase of more accessible surface N-enriched catalytic sites and electron transfer channels inside Fe–N–C catalysts can effectively assure the rapid electron transport rate and further enhance the ORR electrocatalytic activity. 

Herein, we propose a new strategy for easy synthesis of nitrogen-containing carbon nanolayers-decorated CNT nanocomposites (N-C@CNT-Fe) for oxygen reduction electrocatalysis using partially carbonized hemoglobin (Hb) as a single-source carbon and nitrogen precursor, and iron-containing compound as a metal source. The main role of this method is to effectively promote the formation and maximizing exposure of nitrogen-enriched active sites to boost the oxygen reduction activity. The prepared catalyst (N-C@CNT-Fe) shows outstanding ORR electrocatalytic activity as well as better stability compared with a commercial Pt-based catalyst in both alkaline and acidic solutions.

## 2. Materials and Methods

### 2.1. Materials and Chemicals

Hb extracted from porcine blood was purchased from Shanghai EKEAR Bio@Tech Co. Ltd. (Shanghai, China). Multi-walled carbon nanotubes (Electrical conductivity >8000 s/m; Diameter = 10–30 nm) were purchased from Nanjing JCNANO Technology Co. Ltd. (Nanjing, China), and further pretreated in a 5 mol·L^−1^ HNO_3_ solution for 12 h. Other chemicals were from Chengdu KELONG Chemical Reagent Co. (Chengdu, China).

### 2.2. Synthesis of CNT-Based Catalysts

First of all, Hb was partially carbonized at 350 °C for 2 h in flowing-N_2_ atmosphere to produce the Hb350 pyropolymer. Here, the main aim of partial carbonization can promote the decomposition of amino acids and heme structures to be nitrogen-rich or Fe–N moisties, which may facilitate the formation of nitrogen-doped carbon nanolayers on the CNT surface [12,13]. 0.2 g of Hb350, 0.1 g of CNT, and 0.05 g of FeCl_3_·6H_2_O was adequately mixed by a simple ball-milling method for 1 h at 500 rpm. The obtained mixture was further pyrolyzed with a heating-rate of 10 °C·min^−1^ in flowing-nitrogen atmosphere at 800 °C for 2 h. The acquired sample is hereafter called N-C@CNT-Fe. As a control, the N-C@CNT catalyst was similarly prepared by pyrolyzing a mechanical mixture of Hb350 and CNT (mass ratio of 2:1) without addition of metal-Fe. Direct carbonization of Hb at 800 °C for 2 h was used for the preparation of the Hb800 catalyst. Pyrolyzing a simple mixture of Hb and CNT (mass ratio of 2:1) at 800 °C for 2 h was used for the formation of Hb-CNT800. Two-step carbonization of Hb at 350 °C and 800 °C for 2 h was performed to produce the HB350800 catalyst without addition of CNT.

### 2.3. Characterizations and Electrochemical Tests of CNT-Based Catalysts

Shimadzu XRD-6000 X-ray diffractometer (Shimadzu Ltd., Kyoto, Japan) with Cu Ka1 radiation (λ = 1.54 Å) was used to obtain X-ray diffraction patterns with a scanning rate of 4°·min^−1^. Raman spectroscopy was tested with a Renishaw inVia unit (Renishaw Trading Co., Ltd., Shanghai, China) with an excited-λ of 514.5 nm. High-resolution transmission electron microscope (HR-TEM) images were performed on a Zeiss LIBRA 200 FETEM instrument (Carl Zeiss GmbH, Jena, Germany). X-ray photoelectron spectroscopy (XPS) was carried out a VG Scientific ESCALAB 220 iXL spectrometer (VG Scientific, St Leonards-on-Sea, UK) with an Al Kα (*hv* = 1486.69 eV) X-ray source. Electrochemical data were obtained on a Zennium-E workstation (Zahner Elektrik GmbH & CoKG, Kronach, Germany) with a conventional three-electrode device. A glass-carbon rotation disk electrode (GC-RDE, Φ = 4 mm, Model 636-PAR), a saturated calomel electrode (SCE), and a Pt foil with geometric area of 1 cm^2^ were used as working electrode (WE), reference electrode (RE), and auxiliary electrode (AE), respectively. The fabrication of WE refers to our previous reports [13]. The mass loading was controlled to be around 0.40 mg·cm^−2^ except for commercial Pt/C electrocatalyst (20 wt.% Pt, Shanghai Aladdin Bio-Chem Technology Co., Ltd., Shanghai, China) with a mass loading of 0.30 mg·cm^−2^. All potentials (versus SCE) were transformed into the potentials (versus a reversible hydrogen electrode, RHE). All electrochemical experiments were carried out in 0.1 mol·L^−1^ HClO_4_ or KOH electrolytes at a scanning rate of 5 mV·s^−1^. The number of electron transfer (*n*) per O_2_ molecule was calculated by the following equation [16]:

(1) $$1/j_{d}=1/j_{k}+1/B\omega^{1/2}$$

(2) $$B=0.62nFC_{O}D_{O}^{2/3}\nu^{-1/6}\omega^{1/2}$$

where *C_O_* is the O_2_-saturated concentration, *D_O_* is the diffusion coefficient of oxygen molecule, ν is the kinetic viscosity, and ω is the electrode rotation rate.

## 3. Results and Discussion

**Figure 1:**
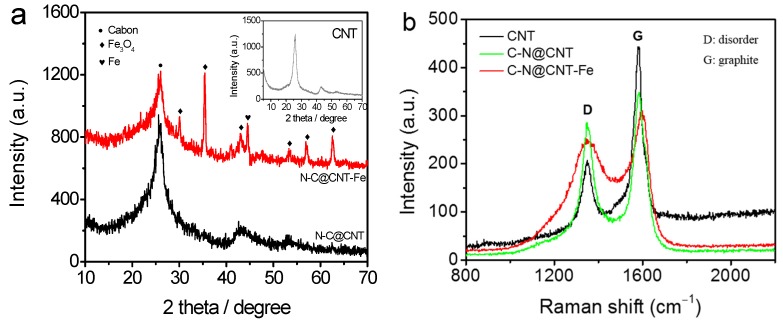
XRD patterns (**a**) and Raman spectra (**b**) of CNT, N-C@CNT, and N-C@CNT-Fe.

Figure 1a shows the X-ray diffraction (XRD) patterns of CNT, N-C@CNT, and N-C@CNT-Fe. For N-C@CNT, two amorphous-carbon peaks with 2θ values of ~25° and ~44° can be identified, which are attributed to the (002) and (101) of graphitic planes, respectively [13]. In addition, a finding is that the width of two diffraction peaks increases largely, compared with the XRD pattern of CNT (inset in Figure 1a), which may be explained by the influence of lower graphitization degree [17,18]. However, the XRD pattern of N-C@CNT-Fe exhibits more complex phase composition, and a series of sharp peaks are displayed at 30.2°, 35.6°, 43.2°, 53.6°, 57.1° and 62.7°, which can be ascribed to crystalline Fe_3_O_4_ phases according to the XRD card (No. 19-0629). Moreover, a notable peak of metallic α-Fe phase (110) is located at ~44.7° in XRD pattern of N-C@CNT-Fe, which is in accordance with the result of the literature [19]. To further study the carbon structure of CNT, N-C@CNT, and N-C@CNT-Fe, Raman spectroscopy data were also employed to analyze their defect sites and disordered structures on the surface. As seen from Figure 1b, all Raman spectra are deconvoluted into two components, and the characteristic “D” and “G” peaks can be found, respectively. According to the previously reported results [13,17], the intensity ratio of D/G (*I*_D_/*I*_G_) is closely related to the amount of structural defects on the catalyst surface. Hence, in the present work, the *I*_D_/*I*_G_ ratios are obtained in N-C@CNT and N-C@CNT-Fe, i.e., 0.78 vs. 0.83, suggesting a lower graphitization degree and more defected structures on the surface of N-C@CNT-Fe, implying the surface doping of more nitrogen atoms on the nitrogen-doped carbon layers. Compared to the *I*_D_/*I*_G_ ratio (0.55) of the CNT, it is also confirmed that the CNT surface has been modified by decomposed products of Hb350 pyropolymer in N-C@CNT and N-C@CNT-Fe. 

**Figure 2:**
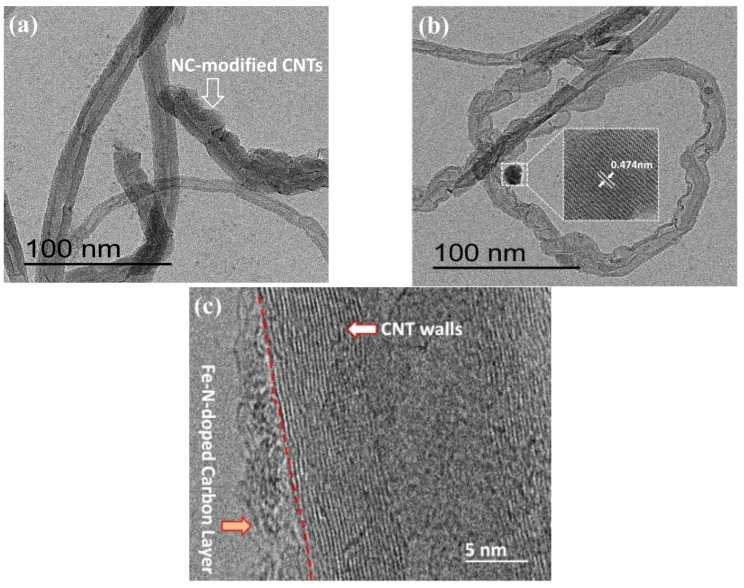
HR-TEM images of N-C@CNT (**a**) and N-C@CNT-Fe (**b**,**c**).

To confirm the above views, the morphology of N-C@CNT and N-C@CNT-Fe was characterized by using HR-TEM technique, and their images were shown in Figure 2. In Figure 2a,b, it can be observed that the surface of partial CNTs is effectively coated with a rough Fe–N–C layer and the thickness of this coating is estimated as 2–5 nm from Figure 2c, which supports the Raman spectroscopy analysis. In addition, the low-resolution TEM image also indicate that N-C@CNT-Fe remain the tubular morphology, although a proportion of CNT support have not been coated by nitrogen-doped carbon layers (see Figure S1). Moreover, Figure 2c clearly displays the graphite layers of CNT and the lattice fringes of Fe–N–C coating. It is worth noting that Fe_3_O_4_ nanoparticle can be observed in Figure 2b and Figure S1, which is in good accordance with the results of XRD analysis. 

**Figure 3:**
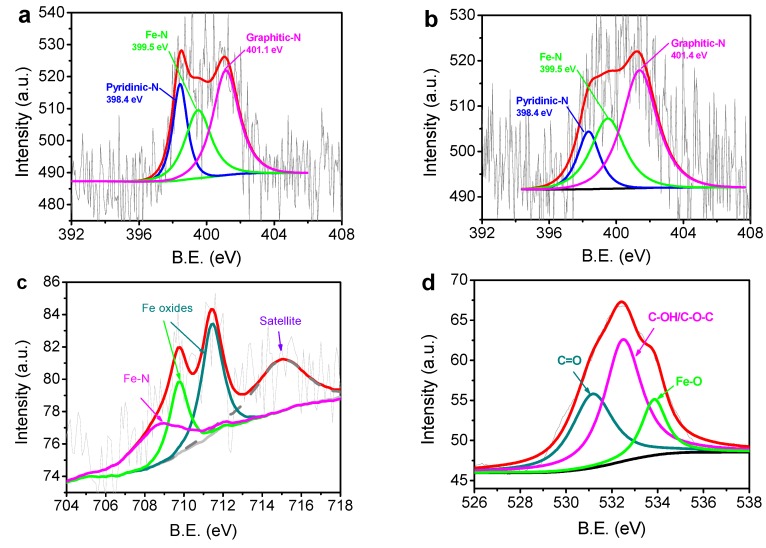
N 1s X-ray photoelectron spectroscopy (XPS) spectra of N-C@CNT (**a**) and N-C@CNT-Fe (**b**); Fe 2p^3/2^ (**c**) and O 1s (**d**) XPS spectra of N-C@CNT-Fe. B.E.: binding energies.

Figure 3 shows the XPS spectra of N-C@CNT and N-C@CNT-Fe. In Figure 3a, the binding energies (B.E.) of three N 1s peaks for N-C@CNT are deconvoluted at 398.4 , 399.5, and 401.1 eV, which can be identified as pyridinic-N, Fe–N, and graphitic-N, respectively [20,21]. For N-C@CNT-Fe, the N 1s XPS spectrum is also split into three peaks with B. E. values of 398.4, 399.5 and 401.4 eV, as indicated in Figure 3b. These results show that the carbon structure of as-prepared catalysts is effectively doped by nitrogen atoms, and the surface N contents are estimated to be 1.55 and 1.27 at. % in N-C@CNT and N-C@CNT-Fe, respectively. The high surface N content of N-C@CNT is further confirmed by high relative intensity and noise-signal ratio of the N 1s spectrum. We also find that high percentages of Fe–N and graphitic-N species can be obtained at two kinds of catalysts, which can be mainly responsible for the ORR electrocatalytic activity. An interesting phenonmenon is that the percentage of Fe–N species in N-C@CNT (~30.0 at. %) is almost equal to that in N-C@CNT-Fe (~30.3 at. %), but a higher percentage of graphitic-N species (~53.2 at. %) can be observed at N-C@CNT-Fe. It effectively indicates that addition of metal-Fe in the precursor has not changed the content of Fe–N species but may facilitate a higher percentage of graphitic-N species to be produced during high-temperature pyrolysis process. To confirm the presence of Fe–N bond in the N 1s XPS spectrum, Fe 2p^3/2^ spectrum is further analyzed in Figure 3c. Except for two iron oxides peaks (710–713 eV) and Fe satellite (714.9 eV), a distinct peak of Fe–N bond is found at 708.7 eV [22,23,24]. XPS survey (Figure S2) of N-C@CNT-Fe also shows that only metal-Fe species can be found without other metal impurities owing to the usage of pure hemoglobin as the precursor. As seen from Figure 3d, the O 1s XPS spectrum corroborates that C=O (531.4 eV), C–OH/C–O–C (532.9 eV), and metal-bounded oxygen (534.1 eV) are formed in N-C@CNT-Fe. Therefore, we consider that Fe–N and Fe–O coexist in the N-C@CNT-Fe catalyst by comprehensive analysis of Fe 2p, N 1s, and O 1s spectra.

**Figure 4:**
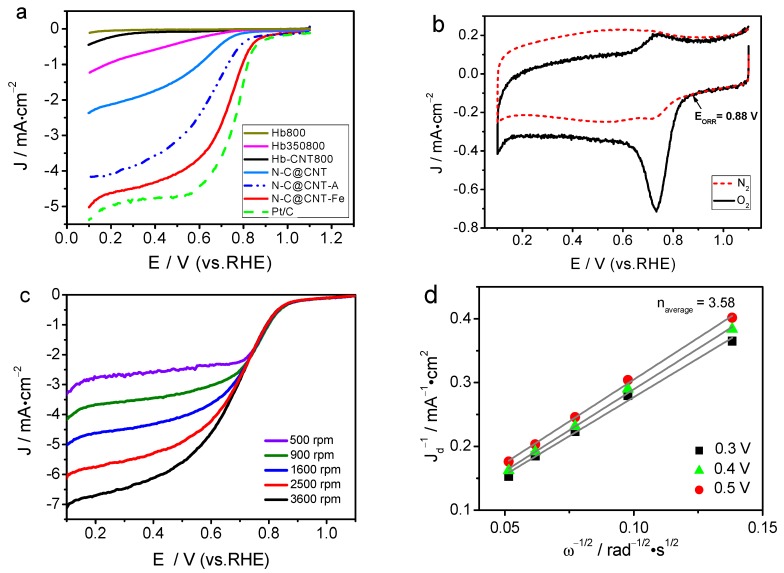
(**a**) Linear sweep voltammetry curves of Hb800, Hb350800, Hb-CNT800, N-C@CNT, N-C@CNT-Fe, N-C@CNT-A, and 20 wt.% Pt/C in O_2_-saturated 0.1 mol·L^−1^ HClO_4_ solution at a rotation rate of 1600 rpm; (**b**) Cyclic voltammetry curve for oxygen reduction reaction (ORR) of N-C@CNT-Fe in O_2_ or N_2_-saturated 0.1 mol·L^−1^ HClO_4_ solution; (**c**) LSV curve for ORR of N-C@CNT-Fe in 0.1 mol·L^−1^ HClO_4_ solution at different rotation rates (500–3600 rpm); (**d**) Koutecky–Levich plots of J^−1^ vs. ω^−1/2^; Data were obtained from (**c**).

The ORR activities of Hb800, Hb350800, Hb-CNT800, N-C@CNT, N-C@CNT-Fe, and 20 wt.% Pt/C were first evaluated by the RDE voltammetry, as shown in Figure 4a. Hb800 exhibits a very low ORR current density and a weak ORR activity. For Hb-CNT800, the ORR current density is slightly improved compared to Hb800. Hb350800 has better ORR activity than Hb800, because the characteristics of carbon matrix and N-doped active sites can be changed by two-step carbonization process [10,11]. N-C@CNT has a relatively higher ORR activity with a half-wave potential (*E*_1/2_) of 0.60 V (vs. RHE) than Hb800, Hb-CNT800, and Hb350800. This result indicates that using the CNT support as a highly conductive agent and inserting matrix to adequately form and expose the catalytically active sites in the carbonization process can greatly enhance its electrocatalytic activity in acidic solution. More importantly, N-C@CNT-Fe exhibits higher positive onset potential (*E*_ORR_) of 0.88 V and *E*_1/2_ of 0.72 V in acidic medium. The difference of *E*_ORR_ and *E*_1/2_ between Pt/C and N-C@CNT-Fe are only 45 and 54 mV. Besides, the limited ORR current density at +0.4 V (vs. RHE) of N-C@CNT-Fe is 4.35 mA·cm^−2^, approach to the value (4.76 mA·cm^−2^) of 20 wt.% Pt/C, and also it is twice as much as that of N-C@CNT (1.73 mA·cm^−2^). In addition, the *E*_1/2_ for ORR measured on N-C@CNT-Fe is higher than those on other NC-based electrocatalysts reported in the literature. That is, the electrocatalytic activity of N-C@CNT-Fe towards the oxygen reduction is comparable to that of the best doped-carbon composite, carbon-based metal-free catalysts, and Fe–N–C catalysts in previously reported literatures [9,10,11,17,25,26,27,28]

The Tafel plots of log (*J*)–*E* curves are shown in Figure S3. The ORR current density is nearly independent with the electrode rotation rate at 0.67–0.87 V (vs. RHE), suggesting that the *J* is mainly dominated by the electrochemical kinetic process. Tafel slopes of N-C@CNT-Fe, N-C@CNT, and Hb350800 are 132.8, 178.4, and 226.0 mV·dec^−1^, respectively. Larger Tafel slopes correspond to a rapid increase in over-potential with current density, resulting in an inferior ORR catalytic activity. The deviation of Tafel slopes for both Hb350800 and N-C@CNT from those for N-C@CNT-Fe implies that their intermediate adsorption process may follow a different model. Besides, the N-C@CNT-A catalyst derived from the treatment of N-C@CNT-Fe in 1 mol·L^−1^ HCl solution for 12 h keeps a relatively higher ORR activity compared to the N-C@CNT catalyst, but its *E*_1/2_ for ORR is 115 mV lower than the untreated N-C@CNT-Fe catalyst. The Tafel slope is also decreased from 132.8 mV dec^−1^ for N-C@CNT-Fe to 172.3 mV·dec^−1^ for N-C@CNT-A owing to the acid-treatment effect, which is attributable to a proper reason that dissolution of metal-Fe can easily occur in the HCl solution, probably leading to partial damage of Fe–N structures and the loss of the ORR activity.

Figure S4 and Figure 4b further show the cyclic voltammetry (CV) curves of N-C@CNT and N-C@CNT-Fe in O_2_- versus N_2_-saturated 0.1 mol·L^−1^ HClO_4_, respectively. Large ORR activity difference can be observed between two catalysts. The CV curve of N-C@CNT exhibits a visible ORR peak at 0.58 V in O_2_-saturated HClO_4_ solution, however, a relatively positive ORR peak shifts to 0.73 V on the CV curve of N-C@CNT-Fe. On the other side, the ORR peak current density of N-C@CNT-Fe approaches twice as large as that of N-C@CNT. Furthermore, both CV curves of N-C@CNT and N-C@CNT-Fe in N_2_-saturated HClO_4_ solution display a virtually featureless current change. These results clearly suggest that CNT support may also play an important role in accelerating charge transfer during ORR in acidic medium, which is supported by our previous study results [12,13,17]. The ORR electrocatalytic behaviors of N-C@CNT-Fe was further analysed by RDE testing at different rotation rates (500–3600 rpm), as shown in Figure 4c. The ORR current density increase with the rotation rate at the catalyzed electrode. Figure 4d shows a good linearity of the Koutecky–Levich (K–L) plots, and suggesting a first-order dependence between ORR kinetics and different potentials (0.3, 0.4 and 0.5 V). The averaged ORR electron transfer number (*n*) is calculated to be 3.58 at different potentials for N-C@CNT-Fe based on the slope of K–L plots. These data indicate that the four-electron reduction pathway occurs during the ORR process of N-C@CNT-Fe, and the electrochemical equation can be expressed as O_2_ + 4H^+^ + 4e^−^ → 2H_2_O. This mechanism is very similar to the ORR electrocatalyzed by a Pt/C catalyst measured in HClO_4_ solution [21].

**Figure 5:**
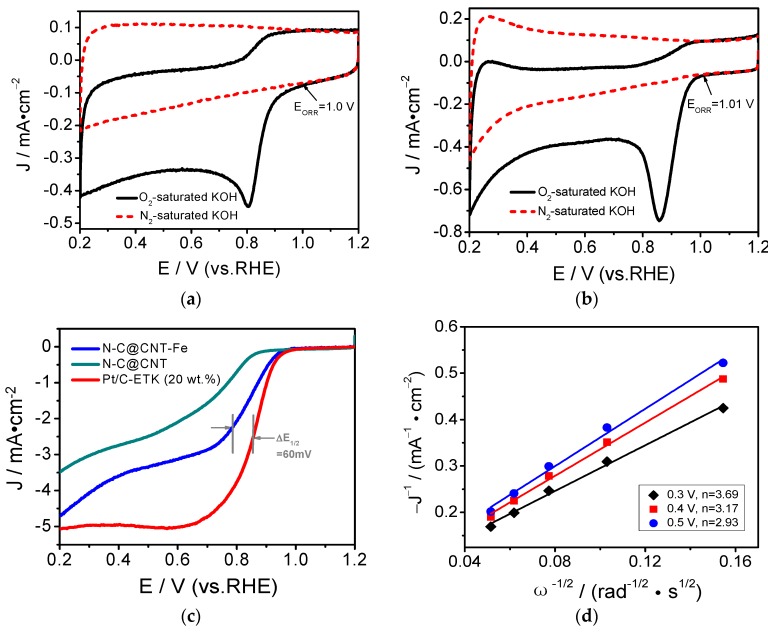
Figure **5.** Cyclic voltammetry curves of N-C@CNT (**a**) and N-C@CNT-Fe; (**b**) in O_2_ or N_2_-saturated 0.1 mol·L^−1^ KOH solution; (**c**) LSV curves of N-C@CNT, N-C@CNT-Fe and 20% Pt/C in O_2_-saturated 0.1 mol·L^−1^ KOH solution at a rotation rate of 1600 rpm; (**d**) Koutecky–Levich plots of J^−1^ vs. ω^−1/2^; Data were obtained from (**c**).

To further investigate the ORR activity of N-C@CNT, N-C@CNT-Fe, and 20 wt.% Pt/C catalysts in 0.1 mol·L^−1^ KOH solution, both CV and RDE methods are carried out and the results are shown in Figure 5. As seen in Figure 5a,b, the ORR onset potentials of N-C@CNT and N-C@CNT-Fe are 1.0 and 1.01 V, respectively. Their *E*_ORR_ are obviously more positive than that in 0.1 mol·L^−1^ HClO_4_ electrolyte. Besides, N-C@CNT and N-C@CNT-Fe have an ORR peak potentials at 0.81 and 0.86 V in O_2_-saturated KOH solution, respectively. However, the ORR peaks are insignificant in N_2_-saturated KOH solution. This also shows that N-C@CNT-Fe has a better ORR activity in the KOH solution because of a small potential difference (∆*E*_1/2_, 60 mV) between N-C@CNT-Fe and 20 wt.% Pt/C (see Figure 5c). In addition, although the limited diffusion current density of N-C@CNT-Fe is lower than that of commercial Pt/C catalyst, N-C@CNT-Fe has larger ORR current density than N-C@CNT. These results clearly indicate that Fe atoms may be a key to the formation of more catalytically active sites for ORR [19], and Fe-doped precursor plays a very important role in the improvement of the ORR activity of final catalyst. Thus, the ORR activity difference between N-C@CNT and N-C@CNT-Fe may be mainly attributed to three aspects: (i) More catalytically active sites are generated for ORR by introducing of CNT into the precursor to prevent agglomeration during pyrolysis [29]; (ii) Electrical conductivity of the catalyst is improved by introducing of CNT during high-temperature pyrolysis, because high conductivity can significantly facilitate to the transport of electrons in the ORR and also enhance the catalytic activity of the carbon-based catalyst [9,21]; (iii) A greater amount of Fe–N and graphitic-N species are obtained in N-C@CNT-Fe compared to N-C@CNT. The ORR catalytic mechanism of N-C@CNT-Fe in alkaline medium was studied by using the RDE at different rotation rates, as shown in Figure S5. The ORR current density increases with the rotation rate on the N-C@CNT-Fe-catalyzed electrode. The good linearity of Koutecky–Levich plots suggests the first-order dependence of the ORR kinetics at given potentials. The averaged electron transfer number of the ORR was calculated to be 3.26 at 0.3–0.5 V versus RHE for N-C@CNT-Fe. According to the slopes of Koutecky–Levich plots, our data indicate that the behavior of ORR on N-C@CNT-Fe is four-electron reduction process, i.e., O_2_ + 2H_2_O + 4e^−^ → 4OH^−^, which is similar to the ORR catalysis of a Pt/C catalyst in KOH solution [30].

**Figure 6:**
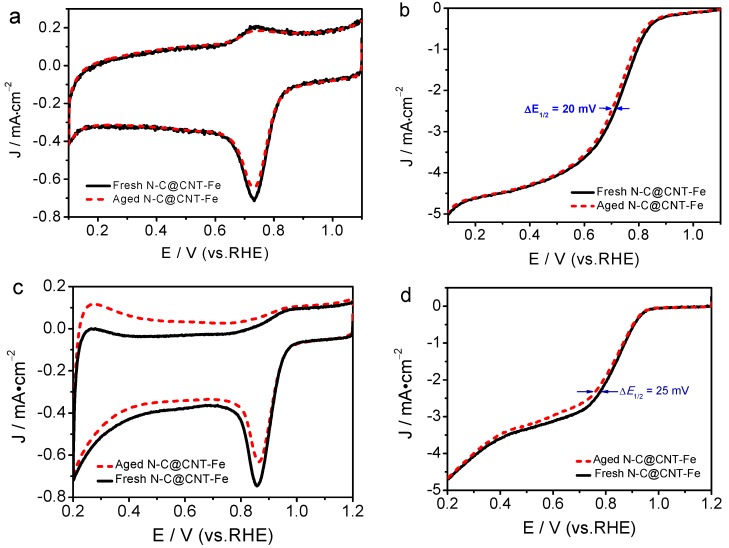
Cyclic voltammetry curves of N-C@CNT-Fe before and after accelerated aging tests (AAT) in O_2_-saturated 0.1 mol·L^−1^ HClO_4_ (**a**) and 0.1 mol·L^−1^ KOH solution (**c**). LSV curves of N-C@CNT-Fe before and after AAT in O_2_-saturated 0.1 mol·L^−1^ HClO_4_ (**b**) and 0.1 mol·L^−1^ KOH solution (**d**) at a rotation rate of 1600 rpm.

The ORR electrocatalytic stability is an important performance indicator to assess the practicability of catalyst for fuel cells. Therefore, the accelerated aging tests (AAT) of N-C@CNT-Fe was performed in O_2_-saturated 0.1 mol·L^−1^ HClO_4_ and 0.1 mol·L^−1^ KOH solutions, respectively. Furthermore, to further evaluate the catalytic activity of N-C@CNT-Fe, CV and LSV measurements were carried out under the same experimental condition. Figure 6a,c shows CV curves of N-C@CNT-Fe before and after AAT treatment, respectively. The results indicate that the CV curves have a slight decrease in ORR peak current density despite in acidic and alkaline solutions. The LSV curves of N-C@CNT-Fe-catalyzed electrode (see Figure 6b,d) have a little changes in the *E*_ORR_, and only *E*_1/2_ of ORR have a negatively shifted of 20 mV (in acidic solution) and 25 mV (in alkaline solution), respectively. CV and LSV data suggest N-C@CNT-Fe has a relatively good stability in both acidic and alkaline electrolytes, compared to the tested results of commercial Pt/C catalysts [19]. In addition, the reason that N-C@CNT-Fe shows relatively poor stability in alkaline solution (vs. in acidic solution) may be attributed to easy occurrence of carbon support corrosion in alkaline condition. In summary, the N-C@CNT-Fe catalyst can be used as a candidate catalyst for fuel cells, and it is a promising nanocomposite to replace the expensive Pt-based electrocatalyst.

## 4. Conclusions

Herein, we report a simple method to synthesize an active and stable N-C@CNT-Fe electrocatalyst from high-conductivity carbon nanotubes wrapped by carbonized hemoglobin-derived Fe–N–C nanolayers, which is promising for four-electron oxygen reduction pathway. Introducing Fe species into the precursor produces more active sites for ORR by preventing agglomeration during pyrolysis, significantly facilitating the fast electron transport of the ORR. Carbonized hemoglobin is found to interact with the surface of CNT in forming these ORR active moieties, most likely consisting of pyridinic-N, Fe–N, and graphitic-N groups as detected by XPS. A greater percentage of Fe–N and graphitic-N can play an important role in the improvement of the electrocatalytic activity. Besides, the addition of the CNT support also helps to improve the electrical conductivity of the final catalyst. Our results show that the prepared N-C@CNT-Fe catalyst is a very promising candidate for commercial Pt-based ORR electrocatalysts. 

## Supplementary Materials

The following are available online at www.mdpi.com/1996-1944/10/5/564/s1, Figure S1: Low-resolution TEM image of N-C@CNT-Fe, Figure S2: XPS survey of N-C@CNT-Fe catalyst, Figure S3: Tafel curves of Hb350800, N-C@CNT, N-C@CNT-Fe and N-C@CNT-A for ORR in O_2_-saturated 0.1 mol·L^−1^ HClO_4_ solution, Figure S4: CV curve of N-C@CNT in O_2_ and N_2_-saturated 0.1 mol·L^−1^ HClO_4_ solution, Figure S5: LSV curves for ORR of N-C@CNT-Fe in 0.1 mol·L^−1^ KOH solution at different rotation rates (400–3600 rpm).

## References

[1] Dai L., Xue Y., Qu L., Choi H.-J., Baek J.-B. (2015). Metal-free catalysts for oxygen reduction reaction. Chem. Rev..

[2] Levy N., Mahammed A., Kosa M., Major D.T., Gross Z., Elbaz L. (2015). Metallocorroles as nonprecious-metal catalysts for oxygen reduction. Angew. Chem. Int. Ed..

[3] Guo C.Z., Liao W.L., Sun L.T., Chen C.G. (2015). Synthesis of non-noble nitrogen-containing catalysts for cathodic oxygen reduction reaction: A critical review. Int. J. Electrochem. Sci..

[4] Wang Q., Zhou Z.-Y., Lai Y.-J., You Y., Liu J.-G., Wu X.-L., Terefe E., Chen C., Song L., Rauf M. (2014). Phenylenediamine-based FeN*_x_*/C catalyst with high activity for oxygen reduction in acid medium and its active-site probing. J. Am. Chem. Soc..

[5] Wu G., More K.L., Johnston C.M., Zelenay P. (2011). High-performance electrocatalysts for oxygen reduction derived from polyaniline, iron, and cobalt. Science.

[6] Guo C., Hu R., Liao W., Li Z., Sun L., Shi D., Li Y., Chen C. (2017). Protein-enriched fish “biowaste” converted to three-dimensional porous carbon nano-network for advanced oxygen reduction electrocatalysis. Electrochim. Acta.

[7] Vij V., Tiwari J.N., Lee W.-G., Yoon T., Kim K.S. (2016). Hemoglobin-carbon nanotube derived noble-metal-free Fe_5_C_2_-based catalyst for highly efficient oxygen reduction reaction. Sci. Rep..

[8] Zhou X., Bai Z., Wu M., Qiao J., Chen Z. (2015). 3-Dimensional porous N-doped graphene foam as a non-precious catalyst for the oxygen reduction reaction. J. Mater. Chem. A.

[9] Nie Y., Xie X., Chen S., Ding W., Qi X., Wang Y., Wang J., Li W., Wei Z., Shao M. (2016). Towards effective utilization of nitrogen-containing active sites: Nitrogen-doped carbon layers wrapped CNTs electrocatalysts for superior oxygen reduction. Electrochim. Acta.

[10] Guo C., Chen C., Luo Z. (2013). The structural changes of blood pyropolymers and their beneficial electrocatalytic activity toward oxygen reduction. Chin. Sci. Bull..

[11] Guo C., Liao W., Chen C. (2014). Fe/N/C catalysts derived from blood protein and their electrocatalytic activity towards the oxygen reduction reaction in acidic solution. Chin. Sci. Bull..

[12] Guo C.-Z., Chen C.-G., Luo Z.-L. (2014). A novel nitrogen-containing electrocatalyst for oxygen reduction reaction from blood protein pyrolysis. J. Power Sources.

[13] Guo C., Liao W., Li Z., Chen C. (2015). Exploration of the catalytically active site structures of animal biomass-modified on cheap carbon nanospheres for oxygen reduction reaction with high activity, stability and methanol-tolerant performance in alkaline medium. Carbon.

[14] Maruyama J., Abe I. (2006). Carbonized hemoglobin functioning as a cathode catalyst for polymer electrolyte fuel cells. Chem. Mater..

[15] Ding Y., Jia W., Zhang H., Li B., Gu Z., Le Y. (2010). Carbonized hemoglobin nanofibers for enhanced H_2_O_2_ detection. Electroanalysis.

[16] Wang S., Yu D., Dai L., Chang D.W., Baek J.-B. (2011). Polyelectrolyte-functionalized graphene as metal-free electrocatalysts for oxygen reduction. ACS Nano.

[17] Guo C., Liao W., Li Z., Sun L., Chen C. (2015). Easy conversion of protein-rich enoki mushroom biomass to a nitrogen-doped carbon nanomaterial as a promising metal-free catalyst for oxygen reduction reaction. Nanoscale.

[18] Liu R., Wu D., Feng X., Muellen K. (2010). Potentiometric sensing of neutral species based on a uniform-sized molecularly imprinted polymer as a receptor. Angew. Chem. Int. Ed..

[19] Guo C.-Z., Liao W.-L., Chen C.-G. (2014). Design of a non-precious metal electrocatalyst for alkaline electrolyte oxygen reduction by using soybean biomass as the nitrogen source of electrocatalytically active center structures. J. Power Sources.

[20] Shui J., Wang M., Du F., Dai L. (2015). N-doped carbon nanomaterials are durable catalysts for oxygen reduction reaction in acidic fuel cells. Sci. Adv..

[21] Ding W., Wei Z., Chen S., Qi X., Yang T., Hu J., Wang D., Wan L.-J., Alvi S.F., Li L. (2013). Space-confinement-induced synthesis of pyridinic- and pyrrolic-nitrogen-doped graphene for the catalysis of oxygen reduction. Angew. Chem. Int. Ed..

[22] Artyushkova K., Walker C., Patterson W., Atanassov P. (2014). Hierarchically structured non-PGM oxygen reduction electrocatalyst based on microemulsion-templated silica and pyrolyzed iron and cyanamide precursors. Electrocatalysis.

[23] Wang Y., Nie Y., Ding W., Chen S.G., Xiong K., Qi X.Q., Zhang Y., Wang J., Wei Z.D. (2015). Unification of catalytic oxygen reduction and hydrogen evolution reactions: Highly dispersive Co nanoparticles encapsulated inside Co and nitrogen co-doped carbon. Chem. Commun..

[24] Tylus U., Jia Q., Strickland K., Ramaswamy N., Serov A., Atanassov P., Mukerjee S. (2014). Elucidating oxygen reduction active sites in pyrolyzed metal–nitrogen coordinated non-precious-metal electrocatalyst systems. J. Phys. Chem. C.

[25] Charreteur F., Ruggeri S., Jaouen F., Dodelet J.P. (2008). Increasing the activity of Fe/N/C catalysts in PEM fuel cell cathodes using carbon blacks with a high-disordered carbon content. Electrochim. Acta.

[26] Schilling T., Bron M. (2008). Oxygen reduction at Fe–N-modified multi-walled carbon nanotubes in acidic electrolyte. Electrochim. Acta.

[27] Tsai C.-W., Chen H.M., Liu R.-S., Asakura K., Zhang L., Zhang J., Lo M.-Y., Peng Y.-M. (2011). Carbon incorporated FeN/C electrocatalyst for oxygen reduction enhancement in direct methanol fuel cells: X-ray absorption approach to local structures. Electrochim. Acta.

[28] Liu G.C.-K., Dahn J.R. (2008). Fe–N–C oxygen reduction catalysts supported on vertically aligned carbon nanotubes. Appl. Catal. A.

[29] Onodera T., Suzuki S., Mizukami T., Kanzaki H. (2011). Enhancement of oxygen reduction activity with addition of carbon support for non-precious metal nitrogen doped carbon catalyst. J. Power Sources.

[30] Fu X., Liu Y., Cao X., Jin J., Liu Q., Zhang J. (2013). FeCo–N*_x_* embedded graphene as high performance catalysts for oxygen reduction reaction. J. Appl. Catal. B.

