# Supplementary material for: Surface Modification of Multi-Walled Carbon Nanotubes via Hemoglobin-Derived Iron and Nitrogen-Rich Carbon Nanolayers for the Electrocatalysis of Oxygen Reduction
Source: Materials (Basel). 2017 May 20;10(5):564. doi: 10.3390/ma10050564 (PMC5459010; doi:10.3390/ma10050564)
Supplement: Supplementary file 1 [file materials-10-00564-s001.pdf]

Supplementary Materials

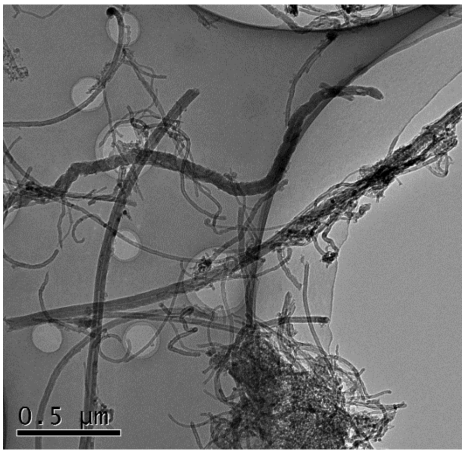

Figure S1. Low-resolution TEM image of N-C@CNT-Fe.

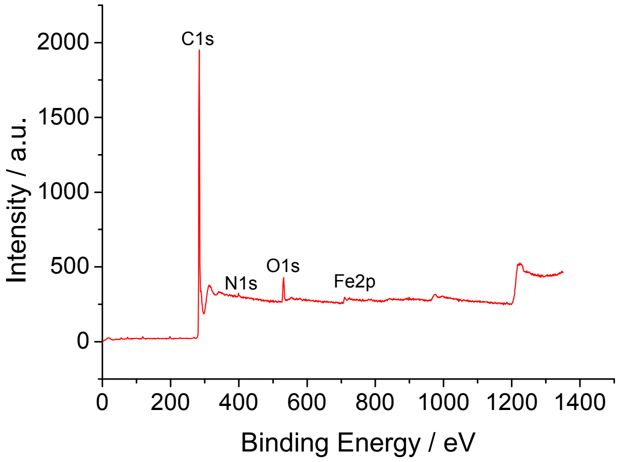

Figure S2. XPS survey of N-C@CNT-Fe catalyst.

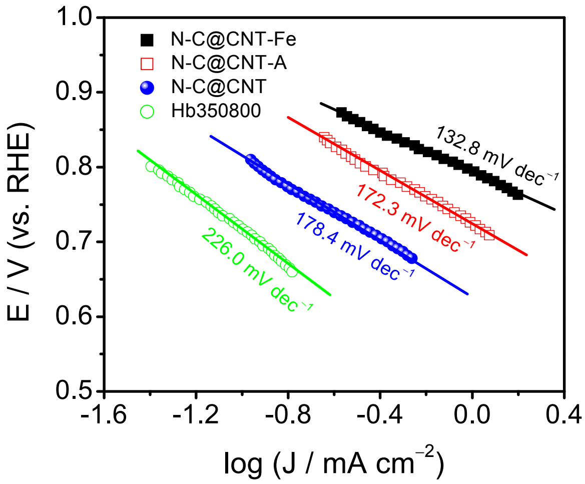

Figure S3. Tafel curves of Hb350800, N-C@CNT, N-C@CNT-Fe and N-C@CNT-A for ORR in O_2-saturated 0.1 mol l^{-1} $HClO_4$ solution.

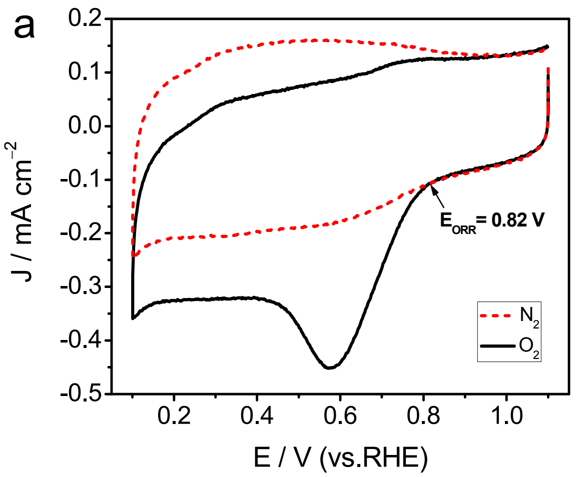

Figure S4. CV curve of N-C@CNT in O_2 and N_2-saturated 0.1 mol l^{-1} $HClO_4$ solution.

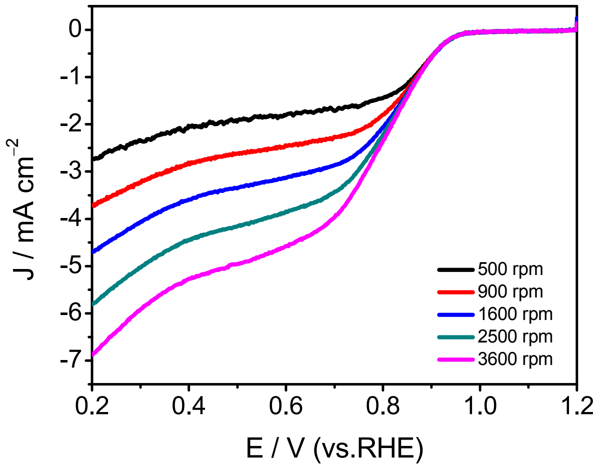

Figure S5. LSV curves for ORR of N-C@CNT-Fe in 0.1 mol l^{-1} KOH solution at different rotation rates (400–3600 rpm).
